# Telerehabilitation for Managing Daily Participation among Breast Cancer Survivors during COVID-19: A Feasibility Study

**DOI:** 10.3390/jcm11041022

**Published:** 2022-02-16

**Authors:** Khawla Loubani, Naomi Schreuer, Rachel Kizony

**Affiliations:** 1 Department of Occupational Therapy, Faculty of Social Welfare & Health Sciences, University of Haifa, Haifa 3498838, Israel; nschreuer@univ.haifa.ac.il (N.S.); rkizony@univ.haifa.ac.il (R.K.); 2 Clalit Health Services, Department of Occupational Therapy, Haifa & Western Galilee, Tel Aviv 62098, Israel; 3 Sheba Medical Center, Department of Occupational Therapy, Tel Hashomer, Ramat Gan 52621, Israel

**Keywords:** occupation-based, meaningful activity, breast cancer, self-management, cognitive strategy, physical activity

## Abstract

We aimed to examine the feasibility and impact of a short-term occupation-based telerehabilitation intervention (Managing Participation with Breast Cancer (MaP-BC)) on daily participation, health-related quality-of-life, and breast-cancer-related symptoms and understand women’s perspectives regarding strategies to manage daily participation and symptoms during COVID-19 pandemic. A mixed-methods study (single-arm pre–post with a qualitative component) included 14 women after their primary medical treatment for breast cancer. Women received six weeks of occupation-based intervention using a video-communication. Sessions focused on identifying functional goals and training strategies to manage daily participation. The primary outcome was perceived performance and satisfaction with meaningful activities by the Canadian Occupational Performance Measure (COPM). Secondary outcomes were participation in the Activity Card Sort (ACS), upper-extremity functioning of Disability Arm Shoulder Hand, self-reported symptom severity, executive-functioning, health-related quality of life, and a question regarding strategies used to manage daily participation. Women significantly improved their daily participation in meaningful activities in the COPM, most ACS activity domains, self-reported executive functioning, and health-related-quality-of-life. Qualitative findings revealed three main themes: (1) daily life under the threats of breast cancer and COVID-19, (2) women’s own strategies to overcome challenges, and (3) contribution of the MaP-BC. Providing telerehabilitation during the COVID-19 pandemic is feasible and successful in improving women’s daily participation after breast cancer.

## 1. Introduction

Women with breast cancer cope with decreased daily participation and quality of life (QOL) due to residual symptoms related to the cancer and its medical treatments [1]. These short- and long-term symptoms include physical (e.g., fatigue, pain, nausea, and limited range of motion in the affected upper limb) [2,3]; cognitive, such as difficulties in executive functioning (e.g., planning and problem solving); attention [4]; and processing speed [5,6] challenges. In addition, emotional and psychosocial difficulties, such as fear of cancer recurrence, death [7], anxiety, and depression, have been reported [8]. Therefore, in the past decade, rehabilitation programs aimed at regaining the daily participation of breast cancer survivors have emerged. Specifically, occupation-based interventions were initiated, and initial results point to their feasibility in improving functional outcomes [9,10,11,12,13].

Worldwide, the COVID-19 pandemic caused restrictions in daily participation and “non-urgent” care, including rehabilitation and follow-up services, which might lead to decreased physical and cognitive functioning and psychological stress [14]. In particular, people with cancer appeared to face greater risks than did the general population [15], specifically, the risks from the pandemic threats (e.g., higher risk of contracting the disease due to a weakened immune system) and the increased prevalence of symptoms due to secondary effects of the pandemic, such as lockdowns [16]. Reduced access to medical and rehabilitation services due to the pandemic caused distress and resulted in reduced physical activity and QOL in breast cancer survivors [17,18]. Moreover, it prevented them from receiving the social support needed to return to their previous roles and daily routines [12,19]. Thus, the COVID-19 restrictions created a need for a rapid transition from in-person rehabilitation to virtual care using telemedicine [20,21].

Telerehabilitation refers to the use of technology to provide remote personalized health care [22]. It allows the exchange of data and communication between patients and health care professionals [23,24], enhancing access to professional care delivery throughout the country. In times of pandemic, it appears to be effective and safe, overcoming the patients’ immune risks, such as when using public transportation to and from clinics [25]. Initial evidence supported the feasibility, acceptability, and effectiveness of telerehabilitation to improve the daily participation of breast cancer survivors [10,12]. In addition, its use was found to improve QOL, functional abilities, and symptom management (including those of pain, depression, anxiety, fatigue, cognitive decline, and sexual dysfunction) by patients with cancer [26,27,28,29,30]. Moreover, Lai and colleagues [31] found that providing telerehabilitation via Zoom software was similar to in-person rehabilitation in terms of the average time required to regain baseline function. In addition, participants reported high satisfaction levels from telerehabilitation.

During the first COVID-19 outbreak, Lopez et al. [32] reported that most of their cancer rehabilitation program visits could be delivered from a distance. Patients and clinicians stated that the telerehabilitation mode of therapy was useful for increasing accessibility to services and helping patients self-manage the disease. Nevertheless, we identified a few gaps in the literature. Specifically, studies that examined personalized occupation-based interventions focused on improving participation in meaningful activities among women with breast cancer in their home contexts or explored women’s perceptions of how they manage their daily routines have been scarce [23]. Moreover, managing a chronic condition, such as breast cancer, requires clients’ active engagement in all aspects of care to obtain better health outcomes [33,34]. Therefore, studies should be designed to echo survivors’ perceptions of strategies that helped them maintain daily participation, empower them to self-manage their new condition, and emphasize the need for a collaborative treatment approach (i.e., client-centered). Finally, discussions of group therapy’s value as part of cancer-rehabilitation programs have emphasized the need to maintain this format in telerehabilitation [32], especially during a pandemic with forced social isolation.

To help fill these gaps, this mixed-methods pilot study aimed to (1) investigate the feasibility and impact of a short-term occupation-based individual and group telerehabilitation intervention named: Managing Participation with Breast Cancer (MaP-BC) [10] on participation and breast-cancer-related symptoms of women with breast cancer during the COVID-19 pandemic and (2) evaluate its contribution to their perceived ability to manage their daily lives under the threats of breast cancer (e.g., residual symptoms and immune system deficiency) and the COVID-19 pandemic.

## 2. Materials and Methods

### 2.1. Design

We carried out a mixed-methods quantitative (single-arm pre–post) and qualitative study design.

### 2.2. Participants

Fifteen women who had been diagnosed with breast cancer and completed their primary medical treatment were recruited through social media and nongovernment organizations (one woman withdrew after receiving three sessions, leaving 14 participants). The inclusion criteria were women with invasive carcinoma Stages I through III, who (a) were at least 3 months post-breast cancer surgery (mastectomy or lumpectomy, unilateral or bilateral) with or without axillary dissection, (b) had completed chemotherapy and radiotherapy, and (c) had previously been healthy. The exclusion criterion was any severe disability that would affect daily functioning (e.g., severe neurological or orthopedic conditions) according to self-reports.

The University of Haifa Ethics Committee approved this study (approval number 218/20). All participants signed informed consent forms. The study is registered in the Australian New Zealand Clinical Trials Registry; number [CTRN12621001295831].

### 2.3. Intervention

This current intervention was adapted from the MaP-BC [10], which improved women’s participation in meaningful daily activities and specifically in high-demand physical activities. The original intervention was a 6-week hybrid intervention, consisting of alternating weekly individual in-clinic OT sessions and telerehabilitation sessions (from the woman’s home, using a special training program) for a total of 12 sessions. During COVID-19, the intervention followed the same occupation-based principles but only consisted of online tele individual and small group meetings, and the training was accompanied by available online video clips.

A trained occupational therapist used Zoom Pro to deliver online synchronic sessions (30–45 min each) of one individual and one small-group meeting weekly for 6 weeks for a total of 12 sessions for each woman. An hour before the prescheduled weekly meetings, each participant received a Zoom link via email from the occupational therapist. The individual sessions focused on identifying occupational-focused goals and barriers to participation, generating, training, and providing metacognitive and self-management strategies to manage and improve women’s daily participation, specifically during the pandemic.

According to each woman’s needs, the individual meetings included motor exercises for the upper extremities with the supervision of the occupational therapist. In addition, both the therapist and the woman shared and discussed ideas for adapting the woman’s home and work environments according to her current abilities, functional needs, and limitations. The group sessions included women with similar symptoms, difficulties, and functional needs. During these sessions, women shared examples and experiences of strategies they used to cope with symptoms and difficulties in their daily functioning. Further, they shared ideas and information on how to use environmental resources, such as health and social welfare (e.g., rights) services, adjunct therapies, and support groups.

### 2.4. Tools

The primary outcomes, perceived performance, and satisfaction with performance in meaningful daily activities were measured by the Canadian Occupational Performance Measure (COPM) [35]. The COPM is a semi-structured interview based on the client-centered approach, which measures self-perceptions of performance and satisfaction with performance in several daily activity domains: self-care (e.g., dressing, walking, transportation), productivity (e.g., work, housekeeping, shopping), and leisure (e.g., reading, sports, socialization). The women chose five meaningful activities and rated them on a scale from 1 (low performance or satisfaction) to 10 (high performance or satisfaction). The final scores of the COPM were the average scores of performance and satisfaction with the performance from the five meaningful activities. COPM has been used and found useful in identifying rehabilitation goals and changes in occupational performance in different stages of the illness among patients with cancer [13,36]. Validity and reliability of the COPM have been established in several populations [37] and used as a primary outcome in randomized control trials among people with Parkinson’s disease [38] and cancer [10].

The secondary outcome of retained level of participation was measured using the Activity Card Sort (ACS) [39], which queries participation in 90 activities divided into four domains: instrumental activities of daily living (IADL), social–cultural leisure, low-physical demand, and high-physical demand activities. Retained activity levels (RALs) for total participation and in each domain were calculated compared to past activities (e.g., 5 years ago). In this study, we used the time pre-breast cancer diagnosis as past activities. During this study’s first assessment, participants reported their participation pre-breast cancer diagnosis (reference time), post-breast cancer diagnosis but before COVID-19 (Time 1), and then during the COVID-19 pandemic (pre-intervention; Time 2). During the second assessment, the women reported their current participation (post-intervention; Time 3). The RAL for each time point was calculated by dividing the weighted score of activities (i.e., continue doing = 1; doing less = 0.5; given up = 0) in the relevant time (i.e., Times 1, 2, or 3) by the number of past activities done by the participant (reference time). For example, if a woman performed 10 of the 90 activities in the past (before diagnosis-reference time) and after diagnosis, she gave up two activities, did less than three activities, and did not add new activities, the RAL for this point of time would be 6.5 divided by ten equals 0.65 (5 activities she kept doing, each scored 1 point = 5, three activities she did less, each scored 0.5 points = 1.5 and two activities she gave up, each scored 0 points–altogether 6.5 points). The ACS was used to measure activity engagement after cancer in community-based survivors [40] and as an outcome measure to assess the feasibility of occupation-focused interventions for women after breast cancer [10,13].

Health-related quality of life (HRQOL) and functional ability were assessed using the Functional Assessment of Cancer Therapy—Breast (FACT-B, 4th ed.) Hebrew version [41]. The tool included 37 questions with responses given on a 5-point Likert scale (from 0 = not at all; to 4 = very much) that constitute five dimensions of wellbeing: Physical, Social/Family, Emotional, Functional, and Additional Concerns. In the current study, we used the total score that was the sum of all the dimensions, ranging from 0 to 148, with higher scores indicating better HRQOL [42]. The FACT-B has good reliability, validity, and internal consistencies [41].

Symptom severity was assessed by using the self-reported symptom severity questionnaire. The questionnaire was developed based on the authors’ clinical experience and qualitative research in people with cancer and has been used in two previous studies [43,44]. Participants ranked perceived symptoms’ severity from 0 (no symptoms) to 4 (severe symptoms). The list of symptoms included physical (pain, fatigue, weakness, peripheral neuropathy), limitations in range of motion (LROM) (upper-extremity on the side of the affected breast), cognitive (memory/attention deficit), and emotional (depression, anxiety, decreased self-perception).

Upper-extremity functioning was assessed with the Disability of Arm Shoulder Hand—Quick version (Quick-DASH) [45], in which lower scores reflect a higher disability. The tool includes 11 items that are rated on a five-point ordinal scale (1 = no difficulty; 5 = unable). Participants were asked to rate the disability and symptoms’ severity experienced by them while performing tasks during the past week. The final score was calculated according to a scoring formula: (([Sum of n responses/n] − 1) × 25), in which n is the number of completed responses. Higher scores indicate a higher disability ranging from 0 to 100. The Quick-DASH has been found reliable and valid for assessing upper extremity disability after breast cancer [46].

Cognitive functioning was tested by the Behavior Rating Inventory of Executive Function—Adult version (BRIEF-A) [47], whose responses were calculated to provide a global executive composite (GEC) score of two index scores: the behavioral regulation index (BRI) and the metacognitive index (MI). The behavioral regulation index consists of four scales (inhibit, shift, emotional control, and self-monitor), and the metacognitive index consists of five scales (initiate, working memory, plan/organize, task monitor, and organization of materials). The BRIEF-A scores are converted to t scores (scores above 65 indicate a cognitive deficit). In the current study, we used the GEC score as well as the two subscales of the tool (i.e., BRI and MI), which represent the women’s self-perception of their executive functioning. The tool was used to assess executive functioning in chemotherapy-treated breast cancer survivors [48].

Qualitative data were collected to deeply understand women’s experiences regarding their breast cancer-related symptoms and daily participation during COVID-19 and strategies they used to manage their daily lives before and after the intervention. An additional aim was to query regarding the contribution of the MAP-BC intervention to daily participation. The semi-structured interviews focused on three main open-ended questions followed by probes, examples, and clarifications: (1) What are your experiences concerning your daily participation, breast cancer symptoms, and medical treatments in general and specifically during COVID-19? (2) How did you manage your daily life facing multiple risks? Post-intervention interviews added another question: (3) How has the telerehabilitation, Managing Participation with Breast Cancer (MaP-BC) [10], contributed to your daily struggle?

### 2.5. Procedure

An occupational therapist, who did not provide the intervention, administered all assessments via online meetings pre- and post-a MAP-BC intervention, which was carried out after completing chemotherapy and radiotherapy. Data collection was carried out during the COVID-19 pandemic between lockdowns.

### 2.6. Data Analysis

We verified normal distributions through Shapiro–Wilk tests. For normally distributed variables, paired *t*-tests were used to compare the women’s performance with their performance satisfaction (COPM, Quick-DASH, and BRIEF-A scores pre- and postintervention). Further, analyses of variance (ANOVA) repeated measures were used to compare between total RAL (ACS) at the three time-points, and multivariate ANOVA (MANOVA) repeated measures to compare between three ACS domains (IADL, social–cultural leisure, and low-physical demand activities) at the three time points. We followed these with Bonferroni post hoc analyses.

For non-normally-distributed variables, we used Wilcoxon Signed-Rank tests to compare symptom severity and the FACT-B pre- and postintervention. We then used Friedman tests to compare participation in the fourth ACS domain (high-physical demand activities) and Spearman’s rho for correlations between symptom severity and participation.

The qualitative data were transcribed verbatim while assuring participants’ anonymity by using pseudonyms (e.g., in their quotations). The three authors analyzed the data together using a phenomenological approach [49], echoing the women’s experiences and perceptions during the extraction and interpretation of text units of the semi-structured transcribed interviews. We interpreted the findings in a reflective and critical manner to cover both the personal and shared experiences of the interviewed women. Fourteen interviews were analyzed according to 13 categories that were grouped into three main themes [49].

## 3. Results

Table 1 presents participant characteristics. Twelve (86%) participants had been working before their breast cancer diagnoses, half of whom did not return to work during the research. Compliance was high, 14 of 15 participants completed the whole protocol, and no technical problems occurred during the online sessions.

### 3.1. Perceived Performance and Satisfaction with Performance (by COPM)

The women had set a large variety of functional goals (Appendix A). Return to physical activity (e.g., sports, walking, and strengthening upper extremities) was one of the most frequent (11/13 women; 85%) meaningful goals the women mentioned. Table 2 shows the mean COPM scores of five activities and clinically significant improvements (i.e., changes ≥ 2 points) [37,38,50] for each woman, as well as mean scores for the group. Significant improvements were demonstrated in both COPM scores, and 10 of 13 (77%) women improved at least two points in three meaningful activities.

### 3.2. Participation in Daily Activities (by ACS) and HRQOL

Overall models of MANOVA repeated measures, as well as the Friedman test, revealed significant differences between the three time points of the five ACS scores (Table 3).

Differences between pre- and postintervention (Time 2 vs. Time 3):

The pairwise comparisons represented in the right column showed that significant improvements were found in total RAL and three out of four activity domains: IADL and high- and low-physical demand.

Differences between pre-COVID-19 and preintervention, i.e., during COVID-19 (Time 1 vs. Time 2): significant decreases in RALs total score and social–cultural domain score were found, as well as a significant increase in the low-demand leisure domain score.

Differences between pre-COVID and postintervention (Time 1 vs. Time 3): significant improvements in RALs of IADL and high-demand leisure were found; however, the postintervention RAL in social–cultural was still reduced compared to pre-COVID.

Further, significant improvements were found in the women’s HRQOL and functional ability, as measured by the FACT-B (z = −2.796; *p* = 0.05) pre-intervention (Mdn = 93.00; range 83.75 to 104.25) compared with postintervention (Mdn = 100.00; range 92.00 to 118.50).

### 3.3. Symptom Severity

Participants experienced moderate to severe self-reported symptoms and decreased upper-extremity functioning, which did not reduce significantly over time. However, significant improvements in women’s cognitive executive functions were demonstrated (Table 4). The improvement in women’s self-perception of their executive functioning was found in the GEC score as well as in both subscales of the BRIEF-A (BRI and MI).

### 3.4. Qualitative Findings

The main objective of the qualitative interviews was to understand how women experienced and managed the symptoms and their daily difficulties after cancer during COVID-19, before and after the intervention. Three themes emerged from the analysis of the semi-structured interviews that were conducted pre- and postintervention. Examples of selected participant quotations are presented in Appendix B, representing women’s own strategies and those adopted during the MaP-BC single and group sessions to cope with the multiple threats resulting from breast cancer, as well as COVID-19.

**Theme 1.***Daily Life Under Threats of Breast Cancer and COVID-19*.

In the first evaluation (preintervention), the participants described their challenges in pursuing participation and social interactions while experiencing isolation and being homebound with overprotective families and friends. They struggled with symptoms that were revealed in the quantitative data and validated by the qualitative data: pain, fatigue, sleeping problems, hypersensitive and decreased function of the upper extremity, cognitive deficiencies, emotional stress, and depression. All participants described the daily physical and, more importantly, the emotional struggle to pull themselves up from their bed and start their routine. Most of them mentioned challenges coping with high-cognitive demanding activities necessary to manage their family and career life. The COVID-19 pandemic imposed multiple risks and a sense of losing control over their life, especially due to restrictions preventing them from receiving therapy and maintaining participation in activities and social interactions.

**Theme 2.***Using Their Own Strategies to Overcome Challenges*.

The women reframed their situations and daily experiences, looking for ways to “lift themselves up.” They used a strategy of utilizing personal resources, such as emotional and cognitive abilities, to cope with functional difficulties and to feel alive, essential, and helpful to their families. These included reframing the situation, positive thinking, and looking for comforting activities. Additional strategies were finding meaningful activities they could do at home as well as adjusting out-of-home activities to minimize fatigue and effort. A few (6/14; 43%) benefitted from strategies of using external resources, such as support of their families and support groups with other survivors, which were mostly online due to COVID-19 restrictions and its health threats. However, they expressed difficulties finding strategies to change their priorities and routines. As Dana (pseudonym) conveyed, “I’m trying to find hobbies, to do good stuff for myself. However, it is tough. I must admit I haven’t found any strategies that help me 100%, not yet.”

**Theme 3.***The MaP-BC Telerehabilitation Intervention Contribution*.

In the second evaluation, all 14 women focused mostly on the support and strategies they gained during the MaP-BC telerehabilitation intervention. Their responses echoed their ability “to manage” the situation and gain some sense of control over the symptoms, residual medical treatment, and their social and personal roles. They valued the helpful information and strategies to cope with the symptoms and to set, prioritize, and pursue realistic functional goals. They emphasized the legitimacy gained from the therapist and the group to acknowledge their cognitive deficits in front of their families, friends, and employers and to adopt more effective management strategies. The sessions helped the women to accept the current situation and to strive for their goals, despite the COVID-19 lockdown.

## 4. Discussion

This study examined the impact of a short-term occupation-based individual and group telerehabilitation intervention, the MaP-BC, on participation and breast-cancer-related symptoms, as well as the participants’ perceived ability to manage their daily lives under the multiple threats resulting from breast cancer, as well as COVID-19. The tele occupation-based intervention was found to be feasible in terms of participants’ compliance and technological aspects and in terms of achieving its main objectives. Significant improvements in the women’s daily participation were reflected in the quantitative outcome measures and confirmed by the qualitative findings. These were accompanied by significant increases in HRQOL, self-reported executive functioning, and the ability to generate metacognitive and self-management strategies to cope with the challenges of breast cancer and COVID-19. However, they were not accompanied by significant improvements in self-reported physical or emotional symptom severity.

The women’s enhanced participation in the management of their daily activities following breast cancer after the intervention aligns with previous studies [10,11,12,13] but also highlights its efficacy when challenges to maintaining participation increase due to a pandemic. Both the quantitative and qualitative findings reflected the focus on metacognitive strategies in improved executive functioning. The well-established link between metacognitive strategies and daily function suggests that the new strategies women gain to self-manage their meaningful daily activities contribute to their improvements in participation [10,51]. It seemed, according to cognitive rehabilitation approaches [52], that participants generalized the use of these strategies in a broad spectrum of activities (reflected in the ACS comprehensive measure) beyond those stated as the most meaningful goals. Moreover, improvements were also seen in HRQOL in accordance with other studies [9,11,12]. All these indicated generalizations of the intervention’s benefits to various aspects of participation and HRQOL, as also demonstrated in previous studies [10,11,12].

This study’s findings also confirmed that decreased participation occurs due to breast cancer [12,19], but further showed the possible effects of the pandemic lockdown: increased in allowed activities (e.g., low-demand leisure-domain activities, such as watching TV), nonsignificant increases in high-demand leisure-domain activities, and decreases in restricted activities (e.g., social–cultural activities, such as going to restaurants). This finding does not accord with Helm et al.’s [17] study, which reported decreased physical activity in women with breast cancer during the pandemic. This discrepancy could be due to the different restrictions governments imposed in each country, as well as varying weather conditions that may have permitted more informal outdoor activities. The greatest postintervention improvement manifested in the high-physical activity domain, as it had in previous studies [10,12]. The importance of physical activity as a health-promotion activity has been well established and is highly recommended by health professionals, especially for people living with and beyond cancer [53]. Indeed, in this study, most participants prioritized high-physical activity as the most meaningful domain. Taken together, these supported the importance of our findings that showed such improvement is feasible from a short-term telerehabilitation program.

Addressing the qualitative data, women talked about striving to cope with the functional difficulties that arose during the COVID-19 pandemic, especially while they had no social and family support due to the enforced social distancing. In previous studies, women with breast cancer declared external support as the main strategy for coping with the consequences of breast cancer on daily life [19,54]. Therefore, it seemed the COVID-19 restrictions increased the need for professional support. The intervention’s individual and group sessions provided additional strategies for participants to manage their daily challenges and legitimize their changed priorities. Moreover, the opportunity to adopt telerehabilitation (with simple technology, such as Zoom) enabled participants to overcome the barriers of needing special technologies, using public transportation, or waiting in crowded clinics.

Our results revealed no significant improvements in the women’s self-reported upper extremity functioning or physical symptoms. Moreover, the disability level reported in the Quick-DASH was higher in comparison to what had been reported in other studies in women with BC [45], and the change in score was not statistically significant and did not reach the minimal clinically important change [55]. This could be explained by the participants’ limitations in performing a structured exercise program and by the clinicians’ limitations in performing hands-on physical evaluation and training [31,56], as had been conducted in previous studies [10,11]. People living with and beyond cancer can benefit from structured-exercise rehabilitation to re-establish confidence and trust in their bodies and physical abilities [56]. Future teleinterventions provided by a customized telerehabilitation system, such as the CogniMotion tele system (https://www.reabilityonline.com/tele-motion; accessed on 26 January 2021) [57], TeraPlus software [58], or using a hybrid tele- and in-clinic program, may overcome this limitation. Nevertheless, randomized controlled trial studies are needed to enhance evidence-based practice, adoption, and tailoring of telerehabilitation for people living with and beyond cancer [10,11,23].

Moreover, our results revealed no significant improvements in the self-reported emotional symptoms. This could be explained by the uncertainty imposed by the pandemic in terms of its impact on health status and the provision of health services, as well as social distancing, all limiting the availability of support and increasing stress. In addition, the emotional symptoms were assessed by one question; in the future, it is recommended to use a more comprehensive assessment of the emotional status, as well as integrating psychological interventions.

This study used a small sample without a control group, and only women who had access to technology and the Internet were able to take part; therefore, results should be generalized cautiously. Due to COVID-19 restrictions, only self-reported outcome measures were used. Future studies should include additional outcome measures that are performance-based and include a therapist’s observation. In addition, the use of multiple tools may cause results’ bias. Finally, due to the study’s design, we cannot conclude which part of the intervention worked (i.e., individual vs. group or online).

## 5. Conclusions

Providing an occupation-based telerehabilitation program during the COVID-19 pandemic is feasible in terms of compliance and technological aspects. It might improve women’s daily participation after breast cancer while reducing their risk of a COVID-19 infection. Focusing on women’s prioritized activities and tailoring the intervention to their functional needs as well as facilitating the use of strategies may extend improvement in additional daily activities, executive functioning, and HRQOL, despite the lack of improvement in self-reported physical and emotional symptoms. Moreover, we showed that a small group telerehabilitation intervention is possible, broadening women’s repertoire of strategies and practical information. The qualitative data echo lessons the women learned, providing valuable information to be implemented in future interventions. This study may serve as initial evidence and a basis for future studies examining effective telerehabilitation interventions for persons with cancer in times of a pandemic or other crises. The pandemic’s unfortunate restrictions opened an opportunity and showed that telerehabilitation is feasible and may be incorporated with the standard care in other circumstances for people with chronic health conditions. This warrants a change in health policies and the formulation of guidelines and regulations. The evidence from the current study calls for further scientific examination of telerehabilitation services.

## Figures and Tables

**Table 1:** Participants’ personal and breast cancer characteristics (N = 14).

Variable	M (SD)	Range
Age (y)	48.71 (12.32)	29–74
Time since diagnosis (days)	17.50 (8.38)	10–39
Education (y)	15.50 (4.00)	10–22
	**N (%)**	
**Therapies today**		
None		
**Breast cancer stage**		
I	2 (14.30)	
II	4 (28.60)	
III	8 (57.10)	
**Surgery**		
Mastectomy	7 (50.00)	
Lumpectomy	7 (50.00)	
**Adjuvant therapies at time of diagnosis ^a^**		
Chemotherapy	14 (100.00)	
Radiotherapy	14(100.00)	
Hormone therapy	10 (71.00)	

Note: ^a^ Most women received more than one therapy.

**Table 2:** Comparison of women’s perceived performance (PCOPM) and satisfaction with performance (SCOPM) of five meaningful activities between preintervention and postintervention assessments (N = 13).

	Preintervention Mean Scores		Postintervention Mean Scores		Number of Activities (of Five) with Clinically Significant Change >2	
Woman Number	PCOPM	SCOPM	PCOPM	SCOPM	PCOPM	SCOPM
1	1.00	1.00	9.60	10.00	5.00	5.00
2	3.00	2.20	6.80	9.00	4.00	5.00
3	1.80	2.80	3.00	1.80	3.00	0.00
4	5.75	4.25	4.60	7.60	0.00	3.00
5	2.60	2.60	5.80	6.80	3.00	4.00
6	4.00	1.50	6.00	9.00	2.00	3.00
7	4.40	5.20	5.20	5.00	1.00	1.00
8	1.40	1.00	6.60	6.40	5.00	4.00
9	4.00	1.40	6.00	4.20	3.00	4.00
10	4.00	2.20	8.00	8.20	4.00	4.00
11	3.80	1.60	5.40	4.40	3.00	3.00
12	5.60	7.00	7.60	8.00	3.00	2.00
13	4.20	3.40	6.20	7.40	4.00	4.00
Mean PCOPM and SCOPM (N = 13)						
	**M (SD)**		**M (SD)**		***t*-test (df = 12)**	***p*-value**
PCOPM	3.5 (1.48)		6.21 (1.63)		−4.10	0.001
SCOPM	2.78 (1.78)		6.75 (2.33)		−4.82	0.0001

Note: PCOPM, Performance in the Canadian Occupational Performance Measure; SCOPM, satisfaction with performance in the Canadian Occupational Performance Measure. Scores of one participant were lost due to a technical problem.

**Table 3:** Comparisons of participation in daily activities (Retained activity levels, by ACS) between three points of time (N = 14).

Scores (RAL)	After BC before COVID-19 (Time 1)	Preintervention (Time 2)	Postintervention (Time 3)	F (2,26)	P	ηp2	Pairwise Comparison
	**M (SD)**	**M (SD)**	**M (SD)**				
Total activities	0.63 (0.22)	0.59 (0.20)	0.76 (0.21)	15.30	0.0001	0.72	3 > 2 1 > 2
IADL	0.65 (**0.19**)	0.65 (0.18)	0.85 (**0.25**)	11.29	0.0001	0.47	3 > 1 3 > 2
Social–cultural	0.58 (0.30)	0.38 (0.19)	0.48 (0.19)	10.12	0.001	0.44	1 > 2 1 > 3
Low-demand leisure	0.87 (0.31)	0.97 (0.39)	1.12 (0.37)	7.99	0.002	0.38	3 > 2 2 > 1
	**Mdn (IQR)**	**Mdn (IQR)**	**Mdn (IQR)**	**χ2**			
High-demand leisure (N = 13) ^a^	0.29 (0–0.833)	0.50 (0.23–0.63)	0.78 (0.39–0.79)	14	0.001	0.15	3 > 2 3 > 1

Note: BC, Breast cancer; IQR, interquartile range; ACS, activity card sort; RAL, retained activity level measured by the activity card sort; IADL, instrumental activities of daily living; ^a^ One participant did not participate in any high-demand leisure activities.

**Table 4:** Comparisons of self-reported symptoms, upper-extremity function (measured by Quick-DASH), and women’s executive function (by BRIEF-A) between preintervention and postintervention assessments (N = 14).

Symptom	Symptom Severity (Range 0–4)			
	Preintervention Mdn (IQR)	Postintervention Mdn (IQR)	Comparison between Preintervention and Postintervention (Z)	
Physical	4.0 (2.75–4.25)	3.0 (3.00–3.25)	−1.93	
LROM	3.0 (2.75–3.25)	3.0 (2.00–3.00)	-0.83	
Emotional	3.0 (1.75–4.00)	2.5 (1.75–3.00)	−1.22	
Cognitive	3.5 (2.00–5.00)	3.0 (1.75–4.00)	−1.73	
	**M (SD)**	**M (SD)**	***t* test (df = 13)**	***p***
Quick-DASH	48.70 (21.26)	41.07 (17.68)	1.98	0.0690
BRIEF-A				
GEC	65.29 (10.97)	55.71 (10.07)	7.62	0.0001
BRI	59.71 (10.54)	51.00 (8.54)	7.12	0.0001
MI	65.57 (16.04)	56.10 (10.66)	2.77	0.0160

Note: IQR, interquartile range; LROM, limited range of motion; Quick-DASH, Quick version of the Disability of Arm Shoulder Hand; BRIEF-A, Behavior Rating Inventory of Executive Function Adult Version GEC, Global Executive Composite of the BRIEF-A; BRI, behavioral regulation index of BRIEF-A; MI, metacognitive index of BRIEF-A.

## Data Availability

The datasets generated and/or analyzed during the current study are not publicly available due to ethical and privacy considerations but are available from the corresponding author on reasoned request.

## Appendix A

**Participant**	**Activity 1**	**Activity 2**	**Activity 3**	**Activity 4**	**Activity 5**
1	Physical activity: sports	Studying	Work	Using public transportation	Physical activity: walking
2	Swimming and aquatic sports	Cleaning the house	Personal hygiene (shaving)	Cooking	Social participation
3	Cleaning windows	Cleaning dust in high places	Arranging clothes	Cooking	Physical activity: gym
4	Dressing/taking off tight clothes	Preparing food	Dealing with administrative issues related to illness	Outside-home mobility	
5	Cleaning the house	Physical activity: strengthening upper extremity	Sitting for long periods	Showering	Cooking
6	Difficulty getting out due to hot flashes	Finding new hobbies	Physical activity	Cleaning the house	
7	Finding new employment	Playing music	Improving the ability to carry shopping bags	Doing laundry	Cleaning the house
8	Physical activity: Feldenkrais	Physical activity: walking	Going back to work (fears of fatigue and social interaction)	Improving fatigue while mobile outside the house	Improving the ability to arrange the house and especially deal with heavy things
9	Improving motivation to draw	Decreasing fatigue while walking and climbing stairs	Strengthening hands to open a bottle	Strengthening hands to carry things	Reading
10	Personal hygiene (improve upper-extremity range of motion)	Dressing	Cognitive performance needed to work	Finding new employment	Cleaning the house
11	Physical activity: cardiovascular capacity	Physical activity: strengthening upper-extremity muscles	Gardening	Finding new employment	Playing music
12	Improving work ability while dealing with neuropathic pain	Improving cognitive abilities needed to work	Physical activity	Improving attention during low-physical activities (e.g., reading, watching TV)	Improving cognitive abilities needed during conversation
13	Improving attention during work	Physical activity	Handcrafts	Improving attention during reading	Reducing distractions during housework

## Appendix B

Theme 1	Experiencing challenges to pursuing participation and social interactions due to BC symptoms and COVID-19 risks
Coping with emotional symptoms and general effects	“I try all the time, and I don’t allow myself to be sick. My mental struggle is much harder than my physical struggle.” (Dana)
	“I think I would just get into this cloud, get into bed, close myself in, and wake up only when COVID is over. Something like that. But, for better or for worse, I have a kid I’m obligated to, and I have to wake up and be with her.” (Lilly)
	“Finding the ways in which I can manage myself within this illness, despite this helplessness to find a way that will enable me to feel in some kind of control over what I do.” (Keren)
	“You know, on one hand, you want to go out and meet people, and on the other hand, because of COVID and because of your immune system and because of everything your body has been through, you are also afraid of meeting people. So you are right in the middle.” (Dana)
	“I had people helping me to be active. Whenever I was down, I had someone to help me get my drive back; and then I felt a drop when everyone left due to the lockdown. Once I was alone, I sank.” (Doris)
Coping with cognitive symptoms	“I was very worried before COVID time about getting back to the class at college and teaching. I was worried about my stance in front of the class of students, gathering the material and speaking about it, and remembering every detail and example. I remember practicing by myself silently and in the end [due to COVID-19] didn’t need to do all that because I did record lessons on ZOOM.” (Naama)
	“Maintaining some kind of routine helps, practicing yoga and using notes, a calendar, and such strategies to compensate for the memory and attention difficulties.” (Katia)
Coping with physical symptoms	“I try to not be angry all the time. For example, I suffer with pain all the time—such as pain in my arms, numbness, and itchiness. Then [I] would [try] not to say anything when I’m around [my family], so as to not put more pressure on myself that I make them suffer.” (Tania)
	“Physically, yes, at home. I fell at home. Yes, this is a very delicate time, and I cannot distinguish between whether it was because of this sensitive time period, which became even more delicate due to COVID.” (Anat)
Coping with challenges to get treatment and support	“Before COVID, during my illness, I joined a support group for women dealing with breast cancer, and right now, because of COVID, we can’t meet up, and we do it through ZOOM. I don’t like the virtual support. I like being around others, so, for now, I don’t join these meetings.” (Dana)
	“For the first time, during COVID, I was worried about dealing with the radiation therapy.” (Tania)
	“During the chemotherapy, we had decided that we can’t allow anyone home. It was a very tough period, almost a year. Only my mom and sisters came to help, and a few friends helped in outdoor tasks.” (Diana)
Theme 2	Using own strategies to overcome the challenges
Reframing the situation and positive thinking	“But I also changed my mind and told myself that I will make this into a fun experience [going out to get chemotherapy]. I began to see that everybody is on lockdown, while I have an outing to the hospital every day, enjoying the way to the hospital, surrounded by nature in bloom during the spring months.” (Tania)
	“The fact that I have a family, the kids help me ‘lift myself up’ and function. I don’t let myself think of bad things. When someone wants to say something to bring me down, I don’t listen. I try not to take it to heart.” (Lilly)
Setting functional goals and looking for meaningful activities	“What helps me is my willpower. I just ‘toss’ the pain aside and do what needs to be done. I set goals, and I don’t think about anything else.” (Lilly)
	“I am home all day. I cannot move; I also avoid moving. I have nowhere to go—common. I am a high risk, and I am scared to death of all that. So these are my plants [on my balcony]. It gives meaning to feel like I’m doing something, to feel essential.” (Sonia)
	“During COVID, I made fewer lists, but I did make sure and succeeded with setting the alarm [to wake up], signed up to all sorts of activities on ZOOM to maintain some sort of normalcy.” (Maria)
Providing and using social support and therapy	“I also try to do more things—phone calls to ask for advice—and give advice perhaps to friends who are also dealing with it. Perhaps to get some ideas from them.” (Maria)
	“During COVID, I use video calls to speak to a therapist. The rest, for now, I’ve let go, including physiotherapy that also helped me a lot. I just work out at home online, and I receive help from an association that helps spiritually.” (Doris)
	“I think that when I was sick the second time, I surrounded myself with recovering women. I think that was very uplifting for me when I find myself on the giving end: giving advice to others, exchanging recipes, exchanging health advice. I also joined the Run initiative, a group of recovering women that I do activities with, and we exchange experiences and advise each other.” (Sonia)
Theme 3.	The contribution of the MaP-BC telerehabilitation intervention
Gaining helpful information and managing the situation, and enjoy life	“There were things I never knew before, such as the ability to go to lymphatic physiotherapy. Now, I get the treatment, and it helps me greatly. I’ve been having less pain, I can move my hand more easily, and I feel like I can start getting back to my presurgery routine. I am very grateful for my participation in the research.” (Lilly)
	“Managing the situation is the headline, and then I categorize it into parts: the academic situation, the medical situation, the health situation. So I do want to say I benefitted from this [intervention], and I do take things I learned from this into my daily life, managing my schedule and my work if I get back to work, and to leave myself some space to enjoy my beautiful life, and how to enjoy it.” (Tania)
	“The research greatly helped me improve—gave me hope that everything has a solution and helped me understand how to lead a healthier life, how to manage my health.” (Lilly)
Maintaining balanced participation in activities	“I learned that I have to take a break during the afternoon and rest. I also realized that when I take my rests in the living room, I don’t rest at all. When I started going to rest in the bedroom, then I fell asleep” (Orit).
	“And then came K (the occupational therapist) and saved me a little. She got me into some kind of routine that helped me function and gain back some weight, and cook, go on walks, and not to give up. Like some kind of movement.” (Doris)
Empathy and tailor-made goals	“The interesting thing about these meetings with K (the occupational therapist)—she managed to understand my hardships, and we started the research with different goals, but we ended up choosing the goal of managing my pain, and it was wonderful. Her availability and attentiveness always. She helped me be part of this process.” (Diana)
	“I put up some general goals during the first meeting, and together with you, I knew how I should achieve those. Whether it was walking more, finding a friend, finding time to do it where it wasn’t too hot, or whether it was exercising, finding an authorized place and the right hours, or whether it was using public transportation, and how to do it during COVID and do it well and take part in it.” (Tania)
	“The things I did with K [the occupational therapist] also helped me talk through how to manage my daily life, and stuff I have to do. How to incorporate it during COVID lockdown for me. So to think about myself, how I treat myself and make sure I’ll stay OK, that I will feel good.” (Anat)
Accepting the illness and setting priorities according to energy	“How to manage the situation, how to rest, how to manage some rough days. I don’t load myself as if I were guilty. Learning how to accept the situation and learning to deal with it.” (Tania)
	“This whole process with K [the occupational therapist], her support, and how she accepted me. I also managed to accept my illness and deal with it; at the end, we managed it together.” (Diana)
	“I feel she’s helped me learn more strategies and give legitimacy to the things I’m experiencing and to understand that these are the most natural things that every woman would have gone through.” (Dana)
Using strategies to manage the cognitive deficit	“We did all sorts of thinking exercises that gave me ways to better handle organizing my thoughts and assignments. First, it helped me believe again in my ability so long as my head works, and it will really be able to complete the assignments.” (Sonia)
	“I learned during the research that when I write things down, when I have a schedule and I write it down, I can note everything in and it makes it much easier and more organized for me. I also learned not to overload my days.” (Orit)
	“Working with K [the occupational therapist] was meaningful in the cognitive sense—this subject was problematic following my illness (scattered, forgetful) and was improved by my work with her. I got new organizational strategies, for example, to read instructions twice, and I gained self-awareness [of my cognitive deficits].” (Keren)
	“During the meetings with K [the occupational therapist], we worked on our focus and memory hardships. I received all sorts of time management options. I learned how to write down things before I drive to places and to speak out loud where I’m going. A lot of techniques and organizational skills to sort the day; these are mostly the things I gained from this research.” (Silvia)
